# Preoperative Concerns of Older US Adults and Decisions About Elective Surgery

**DOI:** 10.1001/jamanetworkopen.2023.53857

**Published:** 2024-01-30

**Authors:** Nicholas L. Berlin, Matthias Kirch, Dianne C. Singer, Erica Solway, Preeti N. Malani, Jeffrey T. Kullgren

**Affiliations:** 1 Department of Surgery, University of Michigan, Ann Arbor; 2 Department of Internal Medicine, University of Michigan Medical School, Ann Arbor; 3 Child Health Evaluation and Research Center, University of Michigan, Ann Arbor; 4 Institute for Healthcare Policy and Innovation, University of Michigan, Ann Arbor; 5 Veterans Affairs Center for Clinical Management Research, Veterans Affairs Ann Arbor Healthcare System, Ann Arbor, Michigan; 6 School of Public Health, University of Michigan, Ann Arbor

## Abstract

This cross-sectional study examines the preoperative concerns among US adults aged 50 to 80 years who considered elective surgery.

## Introduction

Understanding preoperative concerns of older adults is essential to improving patient-centered surgical care for this growing segment of the US population. Our objectives were to characterize preoperative concerns among older US adults who considered elective surgery and whether these concerns were associated with self-reported decisions to have surgery.

## Methods

This cross-sectional study followed the Strengthening the Reporting of Observational Studies in Epidemiology (STROBE) reporting guideline. The University of Michigan institutional review board reviewed this study and deemed it exempt because it did not involve human participants.

In August 2021, we surveyed a nationally representative sample of US adults aged 50 to 80 years through the University of Michigan National Poll on Heathy Aging using the National Opinion Research Center AmeriSpeak panel. This panel is a probability-based sample designed to be representative of the US household population.^1^ The completion rate was 64% (2110 of 3294 adults). Survey questions were developed based on literature review, content and design experts, and pretesting with respondents. Respondents were asked whether they had considered having elective surgery in the past 5 years, which was defined as a procedure that can be scheduled in advance and not done for an immediate life-threatening health problem. Respondents were then asked to rate how concerned they were about a series of surgery-related factors (eg, pain or discomfort, recovery, out-of-pocket [OOP] costs, and employment). Self-reported sociodemographic information, physical health and mental health, and COVID-19 vaccination status were also available from the survey. All statistics were reported as population level estimates using survey weights designed to reflect population figures from the US Census Bureau. Multivariable logistic regression models were performed to determine associations between concerns and decisions to proceed with surgery. All tests were 2-sided, and statistical significance was set at *P* < .05. All statistical analyses were performed with Stata version 16.1 (StataCorp). Data were analyzed from July to August 2023 and November 2023.

## Results

Among 2110 participants, the sample consisted of 1125 females (weighted percentage, 52.7%) and 985 males (weighted percentage, 47.3%) with a mean (SD) age 63.7 (8.1) years. Overall, 676 adults (weighted percentage, 32.0% of older US adults) reported that they considered having an elective surgery in the prior 5 years and 450 of these individuals (weighted percentage, 66.6%) underwent surgery. The 5 most common elective surgical procedures considered were major joint surgery (eg, hip or knee replacement) (123 [weighted percentage, 18.1%]), eye surgery (eg, cataract surgery) (92 [weighted percentage, 12.4%]), abdominal surgery (eg, hernia repair, gall bladder removal, hysterectomy) (73 [weighted percentage, 10.2%]), cosmetic surgery (52 [weighted percentage, 8.8%]), and foot or leg surgery (44 [weighted percentage, 6.8%]).

Among the 676 individuals who considered elective surgery, 417 individuals (weighted percentage, 64.3%) reported being very concerned or somewhat concerned about pain or discomfort and 379 individuals (weighted percentage, 57.2%) reported concerns about difficulty of recovery (Figure). Older adults were most commonly very concerned about OOP costs (144 [weighted percentage, 22.9%]), exposure to COVID-19 (48 [weighted percentage, 18.9%]), and time needed to be off work (41 [weighted percentage, 20.2%]) (Figure). In multivariable models, individuals with concerns about OOP costs, exposure to COVID-19, and time needed to be off work were less likely to undergo surgery (Table).

**Figure.**
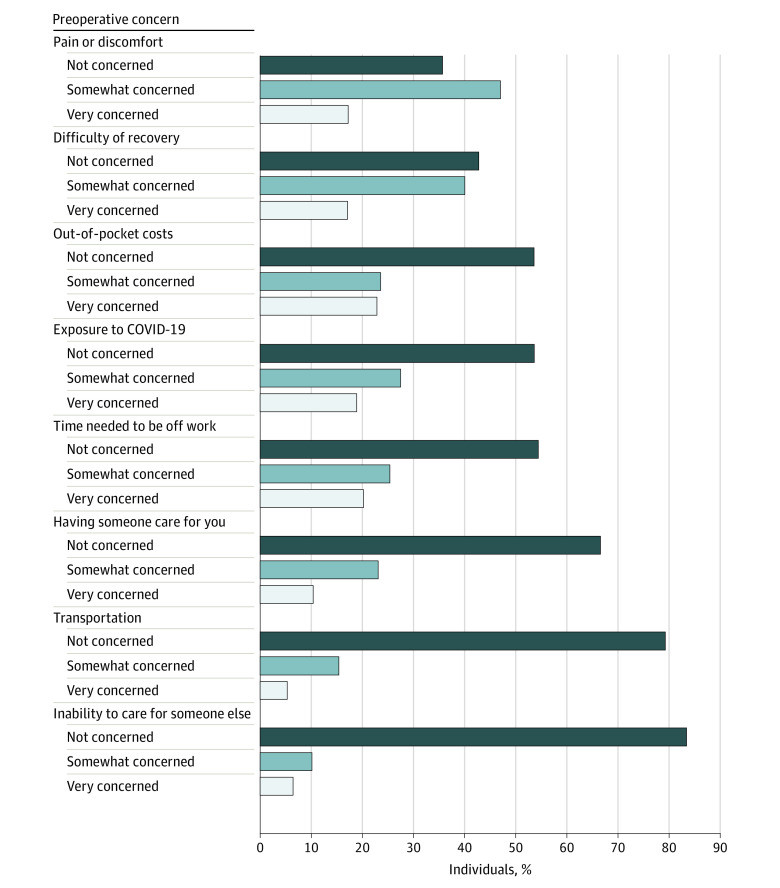
Preoperative Concerns of US Older Adults (Aged 50 to 80 Years) Who Considered Elective Surgery in the Previous 5 Years Values are presented as the adjusted marginal probabilities of each preoperative concern among older adults who considered elective surgery within the past 5 years and weighted using survey weights designed to reflect population figures from the US Census Bureau. These estimates are generated from multivariable logistic regression models adjusted for age, gender, race and ethnicity, education, household income level, self-reported physical health, self-reported mental health, and employment status.

**Table.** Adjusted Associations Between Preoperative Concerns and Self-Reported Decision to Proceed With Surgery^a^

Preoperative concern	Estimated % who had surgery (95% CI), %	*P* value
**Pain or discomfort**		
Very concerned	65.5 (53.3-77.6)	.89
Somewhat concerned	63.7 (57.5-70.0)	.58
Not concerned	66.5 (58.8-74.2)	[Reference]
**Difficulty of recovery**		
Very concerned	56.8 (44.7-69.0)	.07
Somewhat concerned	63.9 (57.0-70.8)	.25
Not concerned	69.6 (62.8-76.3)	[Reference]
**Out-of-pocket costs**		
Very concerned	51.1 (40.6-61.5)	<.001
Somewhat concerned	63.1 (54.2-72.1)	.08
Not concerned	72.5 (66.6-78.5)	[Reference]
**Exposure to COVID-19**		
Very concerned	32.2 (15.1-49.3)	<.001
Somewhat concerned	65.6 (54.7-76.4)	.87
Not concerned	66.7 (58.5-74.9)	[Reference]
**Time needed to be off work**		
Very concerned	39.1 (21.9-56.3)	.01
Somewhat concerned	44.6 (29.8-59.3)	.03
Not concerned	65.1 (55.4-74.7)	[Reference]
**Having someone care for you after surgery**		
Very concerned	52.9 (35.5-70.3)	.10
Somewhat concerned	62.6 (53.5-71.6)	.33
Not concerned	67.8 (62.3-73.3)	[Reference]
**Transportation for surgery and follow-up care**		
Very concerned	70.0 (50.6-89.4)	.70
Somewhat concerned	58.1 (47.3-68.9)	.19
Not concerned	65.9 (60.9-70.9)	[Reference]
**Inability to care for someone else after surgery**		
Very concerned	40.2 (12.2-68.2)	.07
Somewhat concerned	64.2 (51.8-76.6)	.69
Not concerned	66.9 (62.1-71.7)	[Reference]

^a^ Values are presented as the adjusted marginal probabilities of undergoing elective surgery by the levels of each preoperative concern. Estimates are weighted using survey weights designed to reflect population figures from the US Census Bureau and generated from multivariable logistic regression models adjusted for age, gender, race or ethnicity, education, household income level, self-reported physical health, self-reported mental health, and employment status. Each preoperative concern was modeled separately, such that we created 8 separate multivariable models for whether the respondent underwent elective surgery. Each individual model included 1 of the preoperative concerns along with the covariates. This approach was taken to avoid issues of collinearity between the preoperative concerns.

## Discussion

In this cross-sectional study, older US adults who considered elective surgery were often concerned about the physical aspects of surgery in addition to economic consequences (financial and employment status). Addressing these concerns requires systems and policy-level solutions.

The financial burden of surgery has been the focus of recent federal initiatives to improve price transparency and eliminate out-of-network surprise billing.^2,3^ Whether these initiatives will reduce the financial implications of surgery remains unknown. Additionally, our data provide further evidence that concerns about exposure to COVID-19 may have exacerbated issues of access and contributed to pent up demand for care and preventable morbidity associated with surgical disease.^4,5^ Finally, the share of US adults aged 60 years and older who are employed has doubled between 2000 and 2020.^6^ Future studies should seek to clarify the implications of surgery for employment status and job productivity, in addition to identifying workplace policies that facilitate or limit time off for recovery.

This study has limitations. These concerns are retrospective and may not represent all concerns of patients who consider elective surgery. Our analyses did not account for multiple testing and should be considered exploratory. Appropriateness of surgery and whether patients received estimates of OOP costs, COVID-19 risk, or necessary time off from work are unknown. Overall, these findings highlight opportunities to support older adults who consider elective surgery so that these decisions can be based on clinical benefits, risks, and each patient’s goals and preferences.

## Notes

Supplement.
Data Sharing Statement
