# Supplementary material for: Preoperative Concerns of Older US Adults and Decisions About Elective Surgery
Source: JAMA Netw Open. 2024 Jan 30;7(1):e2353857. doi: 10.1001/jamanetworkopen.2023.53857 (PMC10828908; doi:10.1001/jamanetworkopen.2023.53857)
Supplement: Supplement. — Data Sharing Statement [file jamanetwopen-e2353857-s001.pdf]

Data Sharing Statement

Berlin. Preoperative Concerns of Older US Adults and Decisions About Elective Surgery. *JAMA Netw Open*. Published January 30, 2024. doi:10.1001/jamanetworkopen.2023.53857

Data
Data available: Yes
Data types: Deidentified participant data
How to access data: The de-identified data is publicly available in the National Poll for Healthy Aging website (https://www.healthyagingpoll.org)
When available: With publication

Supporting Documents
Document types: None

Additional Information
Who can access the data: Anyone who requests data
Types of analyses: For any purpose
Mechanisms of data availability: It is publicly available
